# Lipopolysaccharide sensitizes the therapeutic response of breast cancer to IAP antagonist

**DOI:** 10.3389/fimmu.2022.906357

**Published:** 2022-08-31

**Authors:** Xin Liu, Jimmy J. Yao, Zhongxuan Chen, Wei Lei, Rong Duan, Zhenqiang Yao

**Affiliations:** ^1^ Department of Pathology and Laboratory Medicine, University of Rochester Medical Center, Rochester, NY, United States; ^2^ Department of Orthopedics, Tianjin Hospital, Tianjin, China; ^3^ School of Engineering, University of Rochester, Rochester, NY, United States; ^4^ Department of Medical Imaging, Henan University First Affiliated Hospital, Kaifeng, China

**Keywords:** lipopolysaccharide, TNF-α, MyD88, Tolllike receptor 4, apoptosis, breast cancer, inhibitor of apoptosis protein

## Abstract

Inhibitor of apoptosis protein (IAP) is a class of E3 ubiquitin ligases functioning to support cancer survival and growth. Many small-molecule IAP antagonists have been developed, aiming to degrade IAP proteins to kill cancer. We have evaluated the effect of lipopolysaccharide (LPS), a component of the bacterial outer membrane, on IAP antagonists in treating breast cancer in a mouse model to guide future clinical trials. We show that LPS promotes IAP antagonist-induced regression of triple-negative breast cancer (TNBC) from MDA-MB-231 cells in immunodeficient mice. IAP antagonists such as SM-164, AT-406, and BV6, do not kill MDA-MB-231 cells alone, but allow LPS to induce cancer cell apoptosis rapidly. The apoptosis caused by LPS plus SM-164 is blocked by toll-like receptor 4 (TLR4) or MyD88 inhibitor, which inhibits LPS-induced TNFα production by the cancer cells. Consistent with this, MDA-MB-231 cell apoptosis induced by LPS plus SM-164 is also blocked by the TNF inhibitor. LPS alone does not kill MDA-MB-231 cells because it markedly increases the protein level of cIAP1/2, which is directly associated with and stabilized by MyD88, an adaptor protein of TLR4. ER^+^ MCF7 breast cancer cells expressing low levels of cIAP1/2 undergo apoptosis in response to SM-164 combined with TNFα but not with LPS. Furthermore, TNFα but not LPS alone inhibits MCF7 cell growth *in vitro*. Consistent with these, LPS combined with SM-164, but not either of them alone, causes regression of ER^+^ breast cancer from MCF7 cells in immunodeficient mice. In summary, LPS sensitizes the therapeutic response of both triple-negative and ER^+^ breast cancer to IAP antagonist therapy by inducing rapid apoptosis of the cancer cells through TLR4- and MyD88-mediated production of TNFα. We conclude that antibiotics that can reduce microbiota-derived LPS should not be used together with an IAP antagonist for cancer therapy.

## Introduction

Breast cancer (BC) accounts for nearly a quarter of all cancers in women worldwide. It is estimated that 287,8500 new cases of invasive BC in women will be diagnosed and 43,250 will die from it in the United States in 2022 (1). Primary BC can be surgically resected. However, the greatest challenge in treating BC is the recurrence and metastasis to other organs after surgery, leading to about 90% of all BC deaths. None of the current therapies effectively prevents or eliminates it. For decades, radiation therapy (RT), chemotherapy (CT), and hormonal therapy have been used to prevent or treat recurrence and metastasis. However, only a small proportion (5% to 10%) of individuals with BC benefit from these adjuvant therapies (2–4). One reason for the poor effects of CT and RT on BC could be that many cancers have already developed micrometastases when the primary cancers are diagnosed (5–7). There is an unmet need to develop a new approach to prevent and/or eliminate recurrence and metastasis.

Caspases, a large family of evolutionarily conserved, aspartate-specific cysteine proteases, are a critical player in the initiation and execution of cellular apoptosis (8, 9). Initially synthesized as inactive pro-caspases, caspases are rapidly cleaved and activated, in response to intrinsic and extrinsic signals, to activate BCL-2 family pro-apoptotic proteins, Bax and Bak, on the outer membrane of mitochondria, leading to the release of cytochrome C from mitochondria to the cytosol (10). As a result, cytochrome C forms a complex with apoptotic protease activating factor 1 and caspase-9, called the apoptosome, to trigger the apoptotic process (10, 11).

Inhibitor of apoptosis proteins (IAPs) is a group of E3 ubiquitin ligases including eight members: cIAP1, cIAP2, NAIP, Survivin, XIAP, Bruce, ILP-2, and Livin (12). IAP proteins are frequently over-expressed in human tumors and promote cancer cell survival (13). The baculoviral IAP repeat (BIR) domain of IAP proteins directly interacts with caspases 3, 7, and 9 to induce their ubiquitin degradation and thus blocks caspase-mediated apoptotic signaling (13–15). In contrast, under the stimulation of apoptosis, an endogenous second mitochondria-derived activator of caspase (SMAC), which is also called DIABLO, in the mitochondria is released to the cytosol, where it binds and degrades IAP proteins, enabling caspases to induce apoptosis (16).

By mimicking the structure of SMAC, many small-molecule IAP antagonists that degrade IAP proteins have been developed, aiming to treat cancers (17, 18). Several of them, such as GDC-0917, LCL161, Debio1143, HGS1029, and TL32711, have been studied in phase I or II clinical trials (19–24). However, none of these IAP antagonists has been approved for clinical use probably because most patients did not benefit from them (19–24). We recently reported that the degradation of IAP proteins alone by a low dose of IAP antagonist, including SM-164 (25), Debio1143 (AT-406) (26), and BV6 (27), does not kill the cancer cells but allows TNFα to rapidly induce BC cell apoptosis (28). This is consistent with the report that IAP antagonist-mediated apoptosis is TNFα dependent (29). The presence of TNFα in the body can explain why a single IAP antagonist is able to eliminate early-stage bone and lung metastases from triple-negative BC (TNBC) in a mouse model, as we reported (28). We also noticed that most of the patients who participated in previous clinical trials had advanced-stage cancers (19–24). These patients might have received standard chemotherapy (SCT), resulting in resistance of cancer to IAP antagonists due to SCT-induced multidrug resistance (28). We have recommended that an IAP antagonist should be given to a patient before neo-adjuvant or adjuvant chemotherapy because the IAP antagonist does not cause multidrug resistance (28). Additional studies are required to identify other possible factors that limit the effect of IAP antagonist therapy to guide future clinical trials.

Lipopolysaccharide (LPS) is a major component of the outer membrane of gram-negative bacteria, contributing greatly to the structural integrity of those bacteria. It is estimated that a healthy human gut contains about 1 kg of bacterial mass and 10 to 50 g of LPS (30). A tiny amount, 1–5 pg/ml, of LPS circulates in the bloodstream of a healthy person without side effects (31). The levels of LPS in the bloodstream are increased in many conditions, such as a high-fat diet, Crohn’s disease (32), atherosclerosis, and diabetes (33). Its level in circulation is largely increased after bacterial infection and can reach up to 300 pg/ml in patients with sepsis and septic shock (34) and even the lethal dose of 1 to 2 μg/ml (35). As a strong inflammatory mediator, LPS not only promotes tumorigenesis (36, 37) but also increases cancer metastasis through NF-κB (38), β1 integrin-mediated cell adhesion (39), and monocyte-induced interactions between cancer and endothelial cells (40). However, LPS also promotes apoptosis of fused hybrid cells of human BC and epithelial cells (41). Our findings in this report show that LPS promotes the growth of MDA-MB-231 TNBC, because of its elevation of cIAP1 and cIAP2 proteins, but not of ER^+^ MCF7 cells expressing a low level of cIAP1/2 in mouse models. Importantly, LPS sensitizes the therapeutic response of both TNBC and ER^+^ BC to IAP antagonist therapy by inducing the cancer cell apoptosis through toll-like receptor 4 (TLR4) and MyD88 stimulated TNFα production. Therefore, use of antibiotics that kill microbiome to reduce the levels of LPS and TNFα together with an IAP antagonist for cancer therapy should be avoided.

## Materials and methods

### Reagents

MyD88 inhibitor 4210 (42) was gifted by Dr. Saikh at the Army Medical Research Institute of Infectious Diseases, Frederick, MD, USA. Purchase information of reagents are listed as follows: APExBIO (Boston, MA, USA), SM-164 (#A8815), AT-406 (#A3019), and BV6 (#B4653); EMD Millipore (Burlington, MA, USA), TLR4 inhibitor TAK-242 (#243984-11-4); eBioscience (San Diego, CA, USA), Annexin V (AnnV) Apoptosis Detection kit including AnnV antibody and propidium iodide (#88-8005-72); R&D Systems (Minneapolis, MN, USA), TNFα (R&D Systems Cat# 210-GMP) and pan cIAP antibody (#MAB3400); Proteintech (Rosemont, IL, USA), MyD88 antibody (#23230-1-AP); Sigma-Aldrich (St. Louis, MO, USA), β-actin antibody (#A5441), Protein Affinity gel (#P6486), Protein G affinity gel (#E3403), and LPS (L2630); and Invitrogen (Carlsbad, CA, USA), human TNF-α ELISA kit (#KHC3011).

### Animal experiments

Immunocompromised NOD/SCID gamma (NSG) mice (NOD.Cg-Prkdcscid Il2rgtm1Wjl/SzJ) originally purchased from Jackson Laboratory were bred in-house. All experimental protocols were approved by the University of Rochester Committee for Animal Resources. All methods used are in accordance with the American Veterinary Medical Association (AVMA) guidelines and regulations.

Cultured TNBC MDA-MB-231 cells at 90% confluence, as we reported previously (28), were harvested following the standard cell culture procedure. The cells were washed with Hanks’ balanced salt solution (HBSS) twice and adjusted cells at 5 × 10^5^ cells/ml; 10 μl (5 × 10^4^) of the cell suspension was injected into the second left mammary fat pad of 3–4-month-old female NSG mice. On the third day, the mice were randomly divided into four groups, 8–10 mice per group, which were intraperitoneally (IP) injected with vehicle, 1 μg of LPS, 3 mg/kg of SM-164 (SM), or their combination, twice a day, for 4 weeks. To test if LPS helps IAP antagonist to treat the established human TNBC, LPS, IAP antagonist (SM-164 or BV6), and their combination were IP injected into the female NSG mice 15 days after the cancer cell implantation to the mammary fat pad when the tumors were visible. Similarly, to test the effect of LPS on IAP antagonist-induced regression of ER^+^ BC, 5 × 10^4^ MCF7 cells were injected into the second left mammary fat pad of 3–4-month-old female NSG mice. The treatment of LPS/IAP antagonist began on days 3 and 15 after cancer injection for 6 and 4 weeks, respectively. The mice were euthanized, and tumors were collected and weighed.

### Apoptosis assay

MDA-MB-231 cells in 60-mm dishes, 2 × 10^5^ per dish, were cultured in a 37°C incubator with 5% CO_2_ for 24 h followed by treatment overnight (16 h) with vehicle, LPS, TNFα, or their combination with different compounds such as SM-164, TLR4 inhibitor TAK-242, or MyD88 inhibitor 4210 (42). The cells collected through 0.25% of trypsin digestion were stained with anti-Annexin V (AnnV) antibody and propidium iodide (PI) for 30 min using AnnV Apoptosis Detection kit. The stained cells were subjected to analysis by flow cytometry. AnnV^+^ early apoptotic cells and AnnV^+^PI^+^ late-stage apoptotic cells were analyzed using FlowJo software. Similarly, the effect of LPS in combination with an IAP antagonist on MCF7 cell apoptosis was tested.

### Western blotting analysis

The cultured MDA-MB-231 cells at sub-confluence in 100-mm dishes were treated with vehicle, SM-164, LPS, or SM-164/LPS −/+ TLR4 inhibitor or MyD88 inhibitor 4210 (42) for 8 h. The cells were lysed with M-PER mammalian protein extraction reagent (Thermo Scientific, Waltham, MA, USA) containing a protease inhibitor cocktail. As we reported recently (43), 20 μg of whole-cell lysate protein was loaded in 10% sodium dodecyl sulfate–polyacrylamide gel electrophoresis (SDS-PAGE) gels and transferred onto polyvinylidene difluoride membranes. Membranes were incubated overnight with primary antibody for MyD88, h/m cIAP pan, or β-actin or GAPDH followed by incubation with horseradish peroxidase-linked secondary antibody (Bio-Rad, Hercules, CA, USA) for 2 h. The membranes were exposed to enhanced chemiluminescence (ECL) substrate, and signals were detected by a Bio-Rad imaging system.

### Immunoprecipitation

Protein measuring 300 μg from MDA-MB-231 cells lysed in M-PER lysis buffer containing protease inhibitor was incubated with 1 μg of MyD88 polyclonal antibody or normal IgG overnight followed by incubation with 80 μl of Protein A/G (1:1) affinity gel at 4°C. After three times of washing with lysis buffer, 60 μl of 2× SDS-Page sample loading buffer was used to elude the proteins bound to the affinity gel. Eluted protein measuring 25 μl from each sample was used to run Western blotting using h/m cIAP pan antibody, as described above.

### ELISA testing of TNFα

The levels of TNFα in the MDA-MB-231 cell culture medium were tested by ELISA, according to the manufacturer’s instructions (Invitrogen).

### Statistics

Descriptive statistics were presented by means and standard deviations for continuous variables. When data distributions were skewed, median and interquartile range were used instead. Comparisons between two groups were analyzed using Student’s two-tailed unpaired *t*-test and comparison among three or more groups by using a one-way analysis of variance followed by Dunnett’s *post-hoc* multiple comparisons. All analyses were performed at a two-tailed 0.05 significance level.

## Results

### Lipopolysaccharide triggers inhibitor of apoptosis protein antagonist induction of MDA-MB-231 breast cancer cell apoptosis

**Figure 1:**
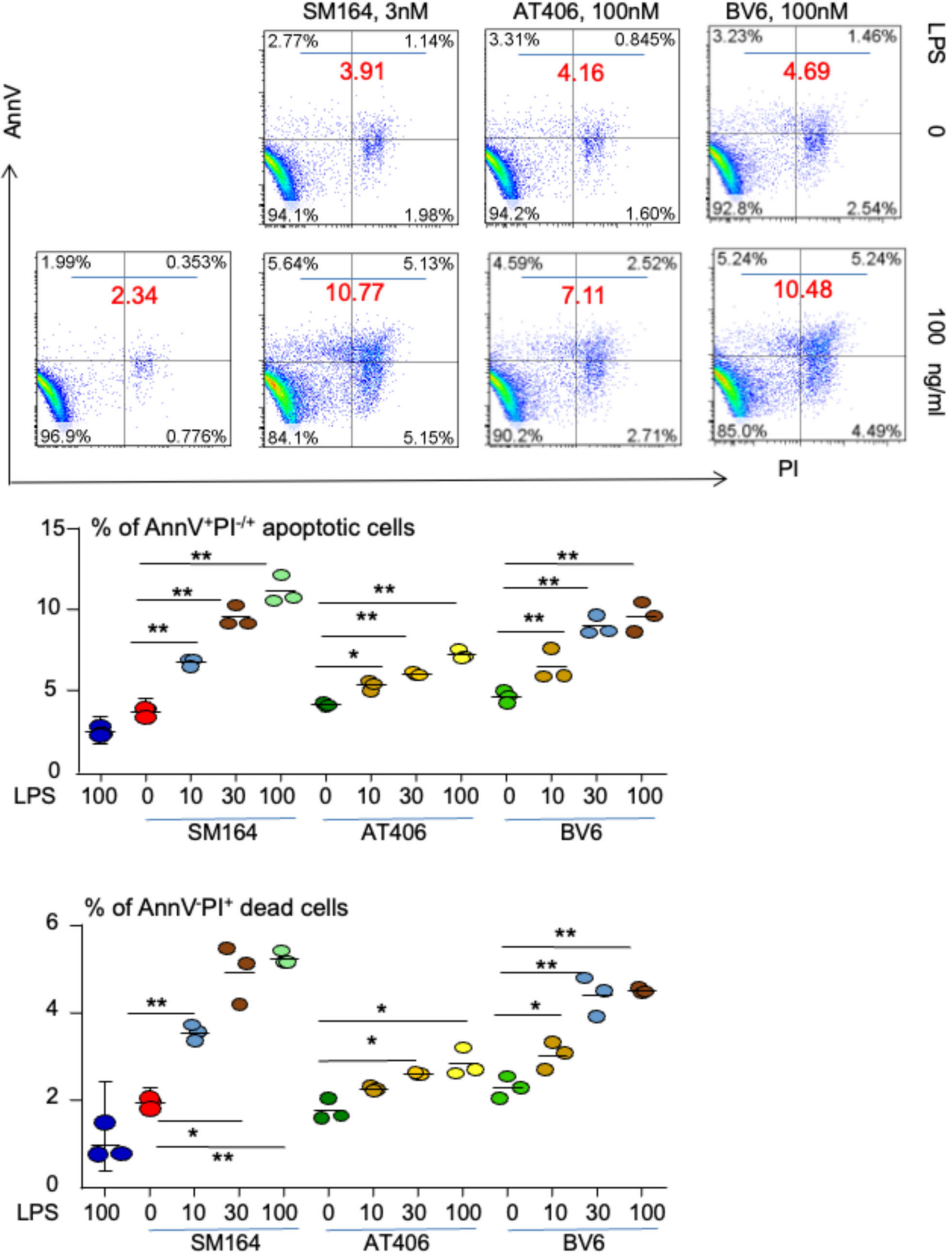
LPS triggers IAP antagonist-induced apoptosis of MDA-MB-231 cells. 1 × 10^5^ MDA-MB-231 cells were cultured in 60-mm dishes for 24 h followed by treatment overnight with indicated dose of SM-164, AT-406, or BV6 −/+ LPS. AnnV^+^PI^−/+^ apoptotic cells were analyzed by flow cytometry. The data were from three repeat samples in one experiment. *p < 0.05 and **p < 0.01 between the indicated groups. The experiments were repeated three times with similar results. LPS, lipopolysaccharide; IAP, inhibitor of apoptosis protein.

TNBC accounts for about 10%–15% of all breast cancer (29), but it is the most aggressive subtype of breast cancer and does not respond to hormone therapy or agent targeted Her-2. MDA-MB-231 TNBC cells were treated with an IAP antagonist, SM-164 (25, 44), AT-406 (45), or BV6 (27), alone or in combination with different doses of LPS overnight (16 h). Low doses of SM-164, AT-406, or BV6 were chosen based on their ability to induce BC cell apoptosis in the presence of TNFα (28). The cells were stained with AnnV and PI to analyze the frequency of apoptotic cells by flow cytometry. Consistent with our recent report that IAP antagonist alone did not induce cancer cell apoptosis (28), the total percentage of AnnV^+^ apoptotic cells treated with SM-164, AT-406, or BV6 was under 5% (not shown). LPS alone, up to 300 ng/ml, did not induce the cancer cell apoptosis either (not shown). In contrast, in the presence of either of these IAP antagonists, LPS, ranging from 10 to 100 ng/ml, markedly and dose-dependently increased AnnV^+^PI^−/+^ apoptotic cancer cell frequency (**Figure 1**). Of note, SM-164 was the strongest one to induce cancer cell apoptosis in the presence of LPS, 3 nM of SM-164 causing more % of AnnV^+^PI^−/+^ apoptotic cells than 100 nM of AT-406 and at least the same % of apoptotic cells as 100 nM of BV6 (**Figure 1**). This is consistent with our recent report that SM-164 is the strongest IAP antagonist to induce cancer cell apoptosis in the presence of TNFα (28).

We also tested if any other factors, except for TNFα and LPS, can trigger the apoptosis of MDA-MB-231 cells in the presence of an IAP antagonist. Unlike TNFα and LPS, none of these factors, including IFN-γ, GM-CSF, IL-1β, IL-4, IL-6, IL-10, and TGFβ1, induced MDA-MB-231 cell apoptosis in the presence of SM-164 (**Supplementary Figure 1**).

### Lipopolysaccharide promotes inhibitor of apoptosis protein antagonist-induced regression of human triple-negative breast cancer in mouse model

We investigated the effect of LPS on the therapeutic outcome of human breast cancer in a mouse model treated with an IAP antagonist. MDA-MB-231 cells were injected into NSG mice orthotopically under the left second nipple. The mice were treated with a low dose of LPS (1 μg) or an IAP antagonist alone or in combination, twice a day, from the third day when the cancer was still undetectable and the 15th day when the cancer was visible, representing insidious and clinically diagnosed stage breast cancer, respectively. A twice-daily dosing regimen was chosen because the plasma half-life of most IAP antagonists is about 4–7 h in humans (20–22, 46), although the half-life of SM-164 and BV6 *in vivo* has not been published.

**Figure 2:**
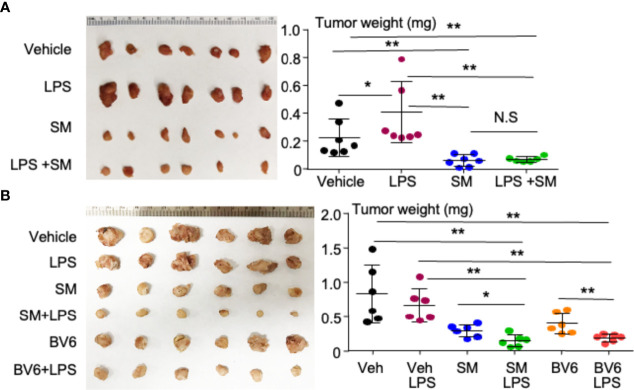
LPS, given together with an IAP antagonist, induces the regression of TNBC in mouse model. **(A)** 2 × 10^4^ MDA-MB-231 cells were injected into the second left mammary fat pad of female NSG mice. From the third day, the mice were treated with vehicle, 1 μg of LPS, 3 mg/kg of SM-164 (SM), or their combination for 4 weeks. The mice were euthanized, and the tumors were collected to scale their weight. **(B)** 2 × 10^5^ MDA-MB- 231 cells were injected into female NSG mice as in panel **(A)**. After 2 weeks, when the tumors were visible, the mice were treated with vehicle, 3 mg/kg of SM, or 10 mg/ml of BV6 alone or in combination with 1 μg of LPS for 3 weeks. The tumor weight was determined as in panel **(A)**. Six to seven in each group. *p < 0.05, **p < 0.01. NS, Not significant. LPS, lipopolysaccharide; IAP, inhibitor of apoptosis protein; TNBC, triple-negative breast cancer.

Starting the treatment at the insidious stage, LPS significantly increased the tumor size compared with vehicle (**Figure 2A**). This is consistent with the reports that LPS not only promotes tumorigenesis (36, 37) but also increases cancer metastasis through NF-κB (38). SM-164 alone, given from the third day, markedly and significantly reduced the tumor size compared with vehicle or LPS (**Figure 2A**). This is consistent with our recent report that SM-164 alone effectively eliminates early-stage, but not advanced-stage, metastatic breast cancer from MDA-MB-231 cells in the mouse model (28). SM-164 plus LPS did not exhibit an additional effect to inhibit tumor growth compared to SM-164 alone (**Figure 2A**) probably because SM-164 alone already had a strong effect to inhibit the growth of early-stage tumors.

When the tumor grew to a visible (clinically detectable) stage, SM-164 alone was still able to reduce the tumor size compared to vehicle or LPS alone (**Figure 2B**). Interestingly, SM-164 plus LPS exhibited a stronger effect than SM-164 alone to inhibit tumor growth (**Figure 2B**). LPS alone did not have any effect on the tumor growth at this advanced stage (**Figure 2B**). Similarly, BV6 alone significantly reduced the tumor size compared to vehicle or LPS alone, and BV6 plus LPS exhibited a stronger effect than BV6 alone to inhibit tumor growth (**Figure 2B**).

### Lipopolysaccharide triggers SM-164-induced MDA-MB-231 cell apoptosis *via* Toll-like receptor 4

**Figure 3:**
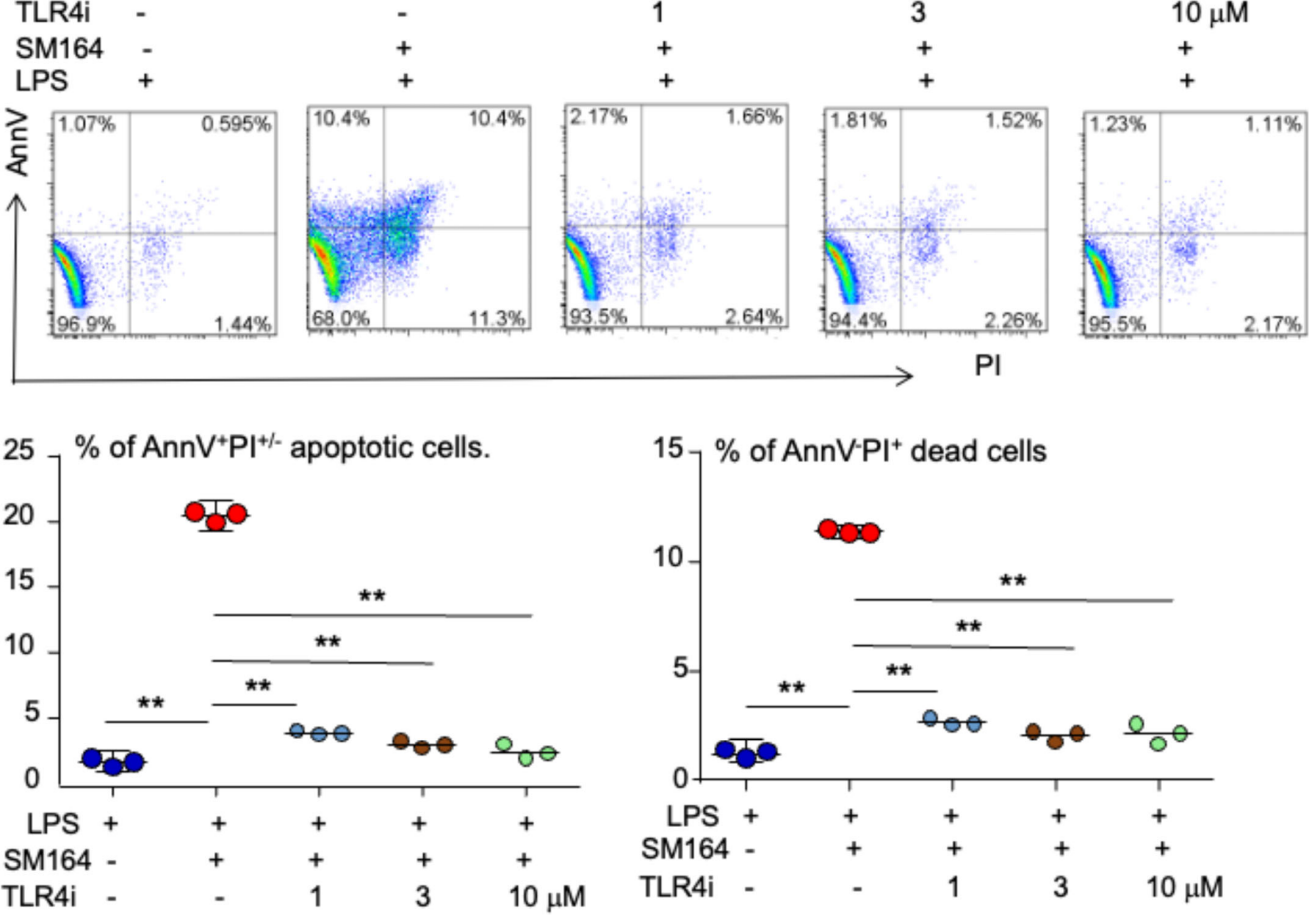
LPS triggers SM-164-induced apoptosis of MDA-MB-231 cells through TLR4. MDA-MB-231 cells were treated overnight with 100 ng/ml of LPS and SM-164 (SM) 3 nM, plus indicated dose of TLR4 inhibitor (TLR4i). AnnV^+^PI^−/+^ apoptotic cells were analyzed by flow cytometry. The data were from three repeat samples in one experiment. **p < 0.01 between the indicated groups. The experiments were repeated three times with similar results. LPS, lipopolysaccharide.

The binding of LPS to transmembrane protein TLR4 recruits its intracellular toll/interleukin-1 receptor (TIR) domain, which associates with the adaptor molecules to activate MyD88-dependent and independent pathways (47). We found that 1 μM of TLR4 inhibitor (TLR4i) almost completely blocked LPS+SM-164 induction of MDA-MB-231 cell apoptosis, the AnnV^+^PI^−/+^ apoptotic cells being reduced by 3.8% *vs*. about 20% of vehicle (**Figure 3**). Increasing the dose of TLR4i did not further impact its effect to block the apoptosis (**Figure 3**) because 1 μM of TLR4 inhibitor had already completely blocked the apoptosis. These suggest that the effective dose of TLR4i is less than 1 μM to block the apoptosis induced by LPS+SM-164.

### MyD88 stabilizes cIAP1/2 but mediates lipopolysaccharide triggering SM-164 induction of MDA-MB-231 cell apoptosis

**Figure 4:**
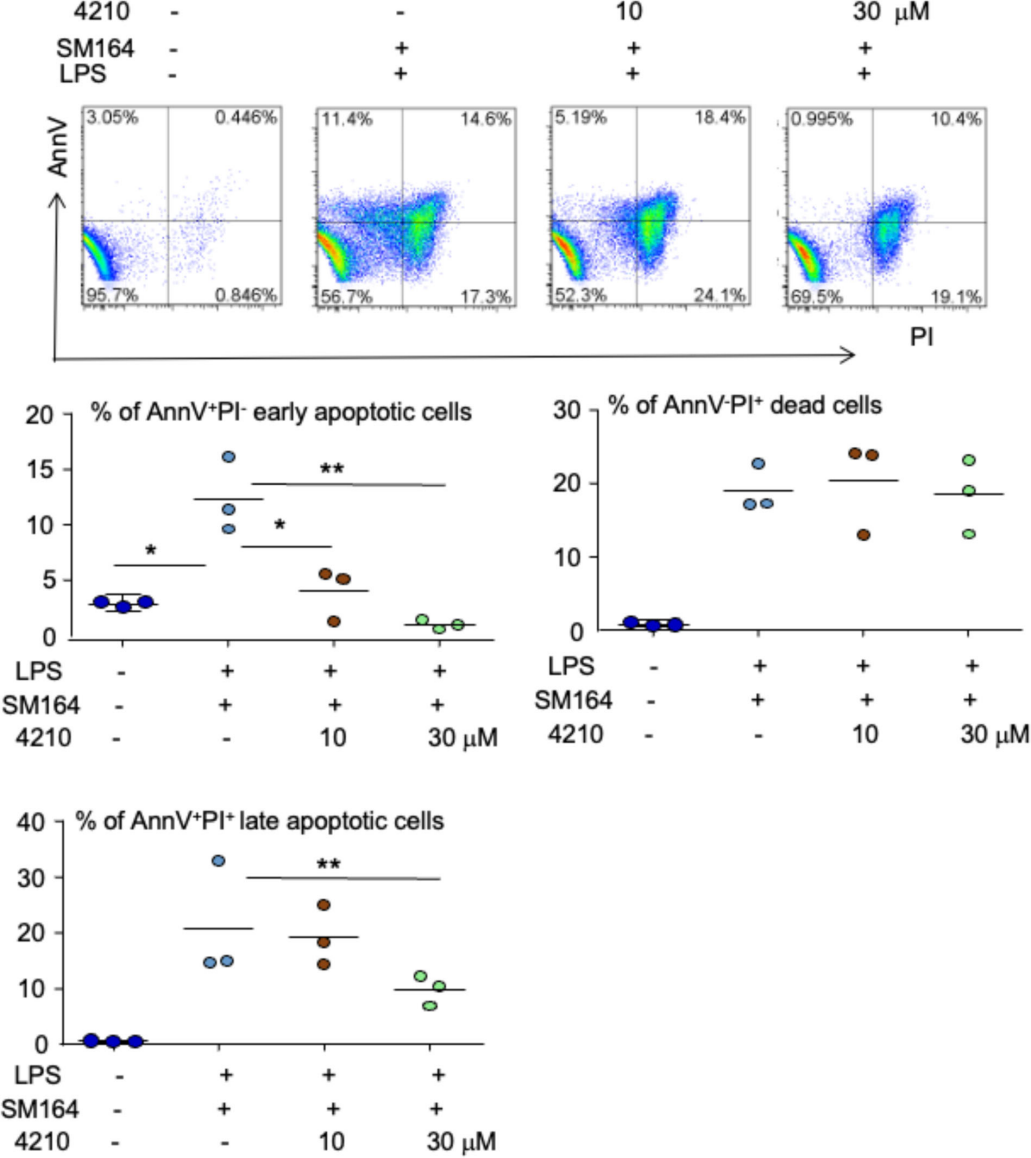
Inhibition of MyD88 blocks LPS triggering SM-164-induced MDA-MB-231 cell apoptosis. 1 × 10^5^ MDA-MB-231 cells were cultured in 60-mm dishes for 24 h. The cells were then treated with vehicle, SM-164 (3 nM) + LPS (100 ng/ml), or SM-164+LPS+ indicated dose of MyD88 inhibitor 4210. AnnV^+^PI^−/+^ apoptotic cells were analyzed by flow cytometry. The data were from three repeat samples in one experiment. *p < 0.05 and **p < 0.01 between the indicated groups. The experiments were repeated three times with similar results. LPS, lipopolysaccharide.

As a canonical adaptor for inflammatory signaling pathways downstream from TLR and IL-1 receptor families, MyD88 links IL-1R or TLR family members to IL-1R-associated kinases (IRAKs) and activates IRAKs *via* homotypic protein–protein interaction, leading to the activation of multiple signaling pathways, including nuclear factor-kappa B (NF-κB), mitogen-activated protein (MAP) kinases, and activator protein 1 (AP-1) (48). To determine if MyD88 transduces LPS/TLR4 signaling to induce cancer apoptosis in the presence of an IAP antagonist, we investigated the effect of a small molecular compound, called 4210, which blocks MyD88 dimerization (42), on the apoptosis of MDA-MB-231 cells induced by LPS+SM-164. The results indicated that 10 μM of 4210 reduced about 50% of AnnV^+^PI^−^ early-stage apoptotic cells induced by LPS combined with SM-164 (5% *vs*. 11%), and 30 μM of 4210 further reduced it to about 1% (**Figure 4**). The compound 4210 also exhibited a trend to reduce AnnV^+^PI^+^ late-stage apoptotic cells induced by LPS+SM-164 (**Figure 4**). However, neither 10 nor 30 μM of 4210 markedly reduced AnnV^−^PI^+^ dead cells (**Figure 4**), suggesting that blocking MyD88 may also cause non-specific death of MDA-MB-231 cells.

**Figure 5:**
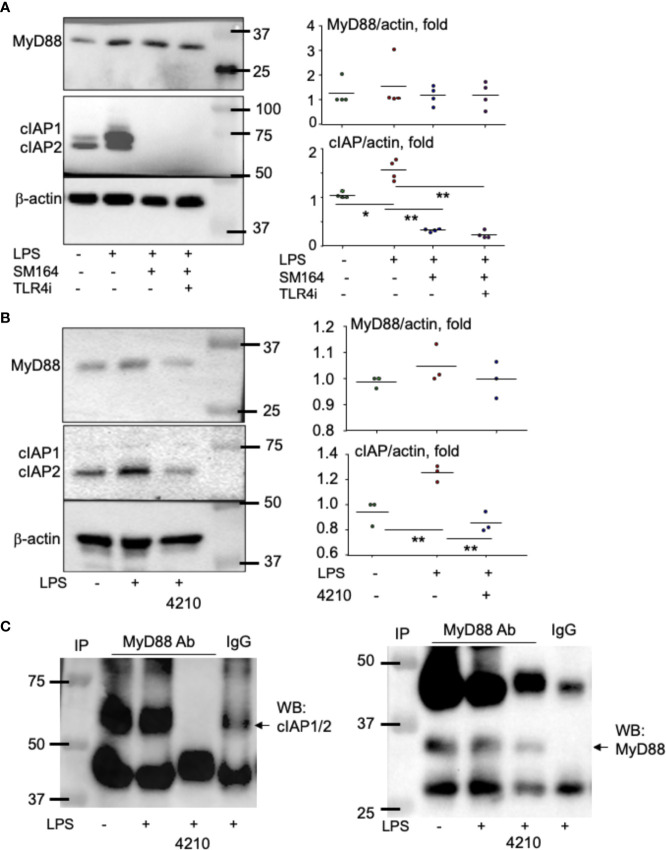
MyD88 associates with and stabilizes cIAP proteins. MDA-MB-231 cells at sub-confluence were treated with LPS (100 ng/ml) or its combination with SM-164 (3 nM) −/+ TLR4i (5 μM) **(A)** and were treated with LPS (100 ng/ml) −/+ MyD88 inhibitor 4210 (10 μM) **(B)** for 8 h; 20 μg of whole-cell lysate protein from each sample was used to test protein levels of MyD88, cIAP1/2, and β-actin by Western blotting (WB). The quantified WB data were from five **(A)** or three **(B)** repeats. *p < 0.05 and **p < 0.01 between the indicated groups. **(C)** Cell lysate protein measuring 300 μg from samples, as in panel B, was immunoprecipitated with anti-MyD88 antibody −/+ 10 μM of MyD88 inhibitor 4210. In addition, one sample was incubated with normal IgG. The pulldown proteins were used to test cIAP1/2 and MyD88 by WB. The experiments were repeated three times with similar results.

To determine how MyD88 mediates LPS-induced cancer cell survival and apoptosis in the presence of an IAP antagonist, we analyzed the expression pattern of the related proteins. MyD88 protein level was not significantly changed, although it was slightly increased in some experiments upon LPS stimulation, and SM-164 alone or its combination with TLR4 inhibitor did not affect MyD88 protein levels (**Figure 5A**). However, LPS strikingly increased protein levels of cIAP1 and 2, which were completely degraded by the addition of SM-164 (**Figure 5A**).

It is known that MyD88 can form a complex with cIAP1/2 together with other molecules including TNFR-associated factor-2 and Src, leading to the activation of p38, c-Jun N-terminal kinases (JNK), and NF-κB in human macrophage (49). Interestingly, the addition of MyD88 inhibitor, 4210, which blocks MyD88 dimerization (42), markedly reduced LPS-induced cIAP1/2 (**Figure 5B**). Immunoprecipitation assay indicates that MyD88 is directly associated with cIAP1/2 protein, and their association was completely blocked by the addition of MyD88 inhibitor 4210 (**Figure 5C**). As a control, MyD88 protein was not detected in IgG-pulled down protein (**Figure 5C**). These findings suggest that MyD88 directly associates with and stabilizes cIAP1/2. Therefore, LPS alone does not cause cancer cell apoptosis, while it does when it is combined with an IAP antagonist, which degrades IAP proteins.

### Lipopolysaccharide induces MDA-MB-231 cell apoptosis through stimulating TNFα

**Figure 6:**
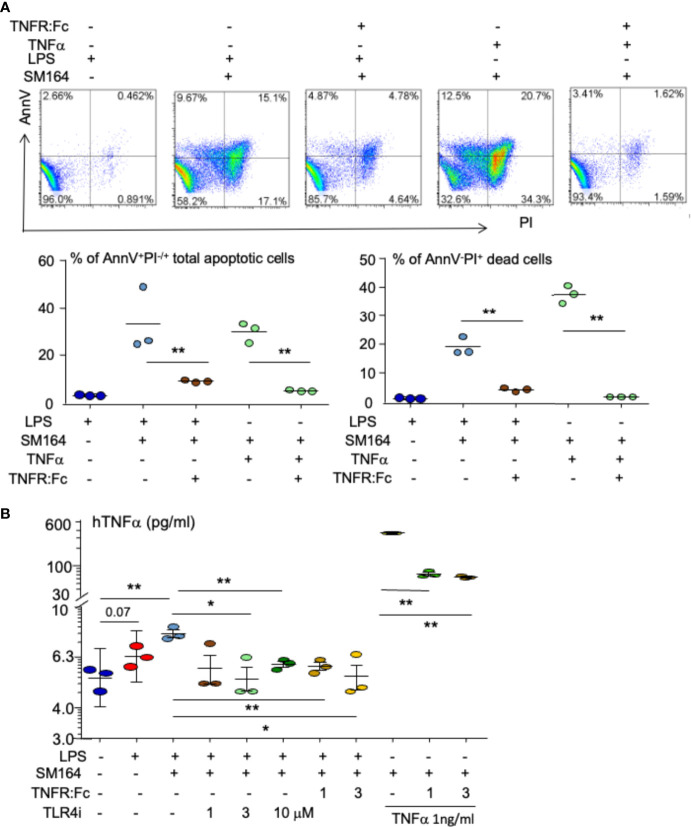
Autocrine TNFα mediated by TLR4 is critical for LPS triggering SM-164-induced apoptosis of MDA-MB-231 cells. **(A)** MDA-MB-231 cells were treated overnight with SM-164 (SM) 3 nM plus either 100 ng/ml of LPS or 1 ng/ml TNFα or with their combination with TNFR: Fc. AnnV^+^PI^−/+^ apoptotic cells were analyzed by flow cytometry. The data were from three repeat samples in one experiment. *p < 0.05 and **p < 0.01 between the indicated groups. **(B)** The culture medium of MDA-MB-231 cells, as in panel A and **Figures 3** and **4**, were collected. TNFα levels were tested by ELISA. The data were from three repeats. *p < 0.05 and **p < 0.01 between the indicated groups. LPS, lipopolysaccharide.

It is known that IAP antagonist induction of cancer apoptosis is TNFα-dependent (27, 28); in particular, the biological function of LPS largely depends on its stimulation of TNFα production (50, 51). We investigated if LPS triggers IAP antagonist-induced BC cell apoptosis through stimulating TNFα. As expected, LPS combined with SM-64 markedly induced MDA-MB-231 cell apoptosis (**Figure 6A**). Addition of TNF receptor IgG Fc segment (TNFR: Fc) (52, 53) decreased AnnV^+^PI^−/+^ apoptotic MDA-MB-231 cells by 9.1% ± 0.5% from 33.3% ± 13% induced by LPS+SM-164 (**Figure 6A**, bottom left panel, p < 0.01). As a positive control, TNFR: Fc decreased TNFα-induced apoptotic cells by 5.1% ± 0.3% from 29.9% ± 4.2% (**Figure 6A**, bottom left panel, p < 0.01). Similarly, TNFR: Fc almost completely blocked AnnV^−^PI^+^ dead MDA-MB-231 cells induced by SM-164 plus either LPS or TNFα (**Figure 6A**, bottom right panel). These findings suggest that LPS stimulates the production of TNFα by the cancer cells to induce the apoptosis of these cancer cells when SM-164 is given together.

The culture medium was collected to test TNFα levels by ELISA. In general, TNFα levels were low in the culture medium (**Figure 6B**) because only a small number of cancer cells were cultured for a short term (overnight). However, stimulation of the cancer cells with LPS markedly increased TNFα levels in the culture medium (**Figure 6B**). The addition of TLR4 inhibitor markedly reduced LPS-induced production of TNFα by the cancer cells (**Figure 6B**), suggesting that LPS-induced TNFα production by the cancer cells is TLR4 dependent. This is consistent with the finding that TLR4 inhibitor blocked LPS-induced MDA-MB-231 cell apoptosis (**Figure 3**). MyD88 inhibitor, 4210, also slightly reduced TNFα levels in the culture medium treated with LPS (**Supplementary Figure 2**), suggesting that LPS stimulates TNFα production by the cancer cells through a MyD88-dependent pathway. Interestingly, TNFR: Fc decreased LPS-induced TNFα levels in the culture medium from MDA-MB-231 cells (**Figure 6B**), suggesting that LPS-stimulated TNFα involves its own production, forming a vicious cycle. This is further supported by the finding that TNFR: Fc also decreased TNFα levels in the culture medium from MDA-MB-231 cells treated with TNFα (**Figure 6B**).

### Lipopolysaccharide combined with an inhibitor of apoptosis protein antagonist induces the regression of ER^+^ human breast cancer expressing low level of cIAP1/2 in mouse model

**Figure 7:**
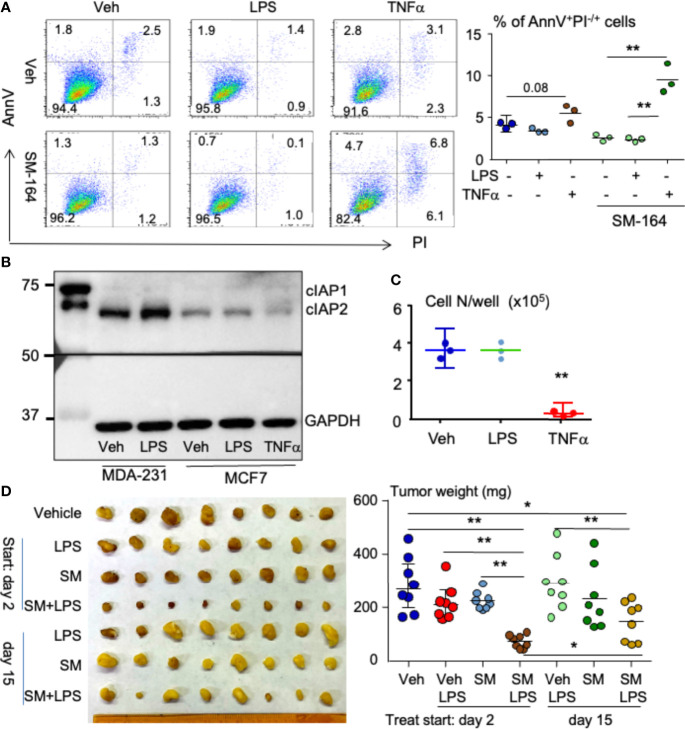
LPS in combination with an IAP antagonist induces ER^+^ breast cancer regression in mouse model. **(A)** 2 × 10^5^ MCF7 cells were cultured in 60-mm dishes for 24 h followed by treatment overnight with vehicle, 100 ng/ml of LPS, or 1 ng/ml of TNFα alone or each of them in combination with 3 nM of SM-164. AnnV^+^PI^−/+^ apoptotic cells were analyzed by flow cytometry. The data were from three repeat samples in one experiment. *p < 0.05 and **p < 0.01 between the indicated groups. **(B)** MDA-MB-231 or MCF7 cells at sub-confluent in 60-mm dishes were treated with vehicle, LPS, and/or TNFα for 8 h; 20 μg of cell lysate protein from each sample was used to test protein levels of cIAP1/2 by WB. **(C)** 4 × 10^4^ MCF7 cells in a well of 6-well plates were treated with vehicle, LPS, or TNFα for 8 days. The cells in each well were digested with 0.25% of trypsin to count cell number. Three repeats for each treatment. **p < 0.01 *vs*. Veh or LPS. **(D)** 2 × 10^5^ MCF7 cells were injected into the second left mammary fat pad of a female NSG mouse. On days 3 and 15 after cancer injection, the mice were treated with vehicle, 1 μg of LPS, 3 mg/kg of SM-164 (SM), or their combination for 4 weeks. The mice were euthanized, and the tumors were collected to scale their weight, eight in each group *p < 0.05, **p < 0.01. LPS, lipopolysaccharide; IAP, inhibitor of apoptosis protein; WB, Western blotting.

About 75% of all breast cancers are ER positive (29). We tested if LPS triggers ER^+^ MCF7 cell apoptosis in the presence of an IAP antagonist *in vitro*. As expected, LPS or SM-164 alone did not cause MCF7 apoptosis (**Figure 7A**). Surprisingly, LPS combined with SM-164 did not cause MCF7 apoptosis either (**Figure 7A**). However, TNFα in combination with SM-164 induced apoptosis of MCF7 cells slightly but significantly (**Figure 7A**). In addition, TNFα alone showed a trend to increase MCF7 cell apoptosis, although the increase of AnnV^+^PI^−/+^ apoptotic cells did not reach statistical significance (**Figure 7A**).

Next, we tested if MCF7 cells failing to undergo apoptosis in response to combined treatment of LPS and SM-164 is related to their expression of IAP proteins. Compared to MDA-MB-231 cells, MCF7 cells expressed low levels of cIAP1 and cIAP2, and the levels of cIAP1 and cIAP2 in MCF7 cells were not increased after either LPS or TNFα treatment (**Figure 7B**). This suggests that the survival or growth of MCF7 cells is independent of IAP proteins. However, SM-164 still enabled TNFα to cause MCF7 cell apoptosis rapidly (**Figure 7A**) probably because SM-164 degrades low levels of IAP proteins.

We also tested if LPS inhibits MCF7 cell proliferation. Unlike TNFα, which significantly and markedly inhibited MCF7 cell growth (**Figure 7C**), as reported previously (54), LPS did not have any effect on MCF7 cell growth (**Figure 7C**).

Finally, we tested if LPS influences the effect of an IAP antagonist in treating ER^+^ breast cancer cells *in vivo*. MCF7 cells were injected into NSG mice orthotopically under the left second nipple. The mice were treated with vehicle, LPS, SM-164, or their combination starting at day 2 when the cancer was still undetectable and day 15 when the cancer was visible, respectively, aiming to determine the effect of LPS/IAP antagonist in treating early- and late-stage ER^+^ breast cancer. Neither LPS nor SM-164 alone influenced the growth of MCF7 cells at the early stage in the mice (**Figure 7D**). This is different from MDA-MB-231 TNBC, whose growth was promoted and inhibited by LPS and SM-164, respectively, at the early stage (**Figure 2A**). However, the tumor size in the mice treated with LPS combined with SM-164 was significantly smaller than that in those mice treated with vehicle, LPS, or SM-164 alone (**Figure 7D**). When the tumor grew at a clinically detectable stage (day 15), treatment of SM-164 or LPS alone did not affect the growth of tumor from MCF7 cells (**Figure 7D**), as expected. In contrast, SM-164 plus LPS slightly but significantly reduced tumor weight compared to vehicle in the mice (**Figure 7D**). However, the tumor weight in mice treated with SM-164 plus LPS starting on day 15 was significantly bigger than in those treated starting on day 3 (**Figure 7D**), suggesting that the effect of SM-164 in combination with LPS in treating ER^+^ breast cancer cells is better when treatments are given earlier.

## Discussion

It is well known that LPS binds TLR4 to recruit downstream signaling and/or adapter molecules, leading to gene expression related to cancer cell proliferation, survival, invasion, and metastasis (55). Our findings in this report first link LPS-mediated breast cancer progression to IAP proteins, although IAP proteins are known to involve in LPS-activated signaling in macrophages (49, 56). Like TNFα (28), LPS markedly increases protein levels of cIAP1 and cIAP2 in MDA-MB-231 cells (**Figures 5A, B**) because MyD88, an adaptor protein of LPS-mediated TLR4, forms a complex with and stabilizes cIAP1 and cIAP2 (**Figure 5C**). As a result, LPS promotes the growth of early-stage human TNBC from MDA-MB-231 cells in the animal model (**Figure 2A**). The concept that LPS promotes cancer progression through IAP proteins is further supported by our findings that LPS alone does not promote the growth of ER^+^ breast cancer (**Figure 7D**) from MCF7 cells expressing a low level of cIAP1/2 (**Figure 7B**).

More importantly, we provide the first evidence showing that LPS sensitizes the therapeutic response of both TNBC and ER^+^ breast cancer to IAP antagonists. IAP proteins are frequently over-expressed in human tumors and promote cancer cell survival by inducing the ubiquitin degradation of caspases 3, 7, and 9 (13–15). Degradation of IAP proteins alone by an IAP antagonist is not sufficient to kill cancer cells (**Figure 1**), as we reported (28), but enables LPS to rapidly induce the apoptosis of cancer cells expressing a high level of cIAP1/2 through TLR4- and MyD88-mediated production of TNFα (**Figures 3**, **4**, **6**), which causes the cancer cell apoptosis when IAP proteins are degraded by an IAP antagonist (28). This is supported by the findings that 1) inhibition of TNFα signaling almost completely block the apoptosis induced by LPS plus IAP antagonist (**Figure 6A**); and 2) inhibition of either TLR4 or MyD88 blocks not only LPS-induced TNFα production by MDA-MB-231 cells (**Figure 6B** and **Supplementary Figure 2**) but also the cancer cell apoptosis induced by LPS plus IAP antagonist (**Figures 3**, ). Interestingly, LPS combined with SM-164 also significantly causes the regression of the ER^+^ breast cancer from MCF7 cells expressing a low level of cIAP1/2 at both early and late stages in the mouse model (**Figure 7D**) because LPS stimulates macrophages to produce TNFα (50, 57), which inhibits MCF7 cell proliferation directly (**Figure 7C**) and also cause its rapid apoptosis in combination with SM-164 (**Figure 7A**). LPS combined with SM-164 does not cause the apoptosis or growth arrest of ER^+^ MCF7 cells *in vitro* probably because MCF7 cells do not produce TNFα even in the presence of LPS. TNFα still causes MCF7 cell apoptosis when it is combined with SM-164 (**Figure 7A**) probably because SM-164 degrades a low level of IAP proteins in MCF7 cells, allowing TNFα-activated signaling, such as NF-κB, to result in tumor cell death (58). Together, LPS-induced regression of breast cancer *in vivo* when IAP proteins are degraded by an IAP antagonist is mainly caused by its stimulating production of TNFα, which rapidly induces cancer cell apoptosis when IAP proteins are degraded.

Macrophage is the main source of TNFα (50, 57) and among the most abundant support cells in a tumor microenvironment (59). Despite IFN-γ, GM-CSF, IL-1β, IL-4, IL-6, IL-10, and TGFβ1 failing to directly induce breast cancer cell death in combination with an IAP antagonist (**Supplementary Figure 1**), it is possible that these cytokines stimulate the production of TNFα by macrophages and T cells to indirectly mediate breast cancer cell death when an IAP antagonist is given. Activated T cells also strongly produce TNFα (60). Immunodeficient NSG mice lack T cells, which may limit the effect of LPS to induce breast cancer regression when it is combined with an IAP antagonist. It will be necessary to test if a minimal level of LPS derived from a normal microbiome is essential for an IAP antagonist to induce the regression of early- and advanced-stage cancer in normal immune conditions.

In a rare case, bacterial infection causes spontaneous regressions of malignancies, including breast cancer, melanomas, renal cell carcinomas, neuroblastomas, and leukemia, without any treatment (61). A strategy of delivering live bacteria for the cure of solid tumors can be traced back more than a century ago to Coley’s toxins (62). Inspired by this approach, scientists have developed modern forms of cancer immunotherapies. One of the most successful ones is Bacillus Calmette-Guerin (BCG), an attenuated *Mycobacterium bovis* strain, which has been approved by Food and Drug Administration (FDA) to treat non-muscle invasive bladder cancer (63). However, scientists still do not fully understand why bacterial treatment works.

Bacterial induction of cancer death is generally attributed to bacteria stimulating the body’s immune system, which destroys invading bacteria while killing malignant cells *via* released cytokines from activated CD4^+^ helper and CD8^+^ cytotoxic T cells (64, 65). Cytokines released in patients with bladder cancer treated with BCG include TRAIL (TNF-related apoptosis-inducing ligand), IL-2, IL-8, IL-18, IL-12, IFN-γ, and TNFα (66). However, each of these cytokines alone does not kill the cancer cells (**Supplementary Figure 1**). Scientists have also developed adoptive T cells expressing chimeric antigen receptors (CARs) that target specific antigens of tumors, which have been approved for clinical treatment of some cancers, especially for pre-B-cell acute lymphoblastic leukemia and diffuse large B-cell lymphoma (67). However, attempts to target tumor-associated antigens in solid tumors have achieved limited success so far (68). Thus, bacteria-killing cancer cells cannot be solely attributed to the activated T cells and the released cytokines. An exception is those cancers expressing low levels of IAP proteins, for example, MCF7 cells, whose growth can be directly inhibited by TNFα (**Figures 7B, C**). Our findings in the mouse model by injecting LPS to mimic bacterial infection provide evidence that bacterial products can directly kill cancer cells to cause the regression of malignancies under specific conditions, for example, degradation of IAP proteins by endogenous SMAC/Diablo (69, 70). However, it is unknown which factor triggers endogenous SMAC-induced IAP degradation to cause regression of the cancers when bacterial infection occurs.

Another possibility is that structural change or defect of IAP proteins in the cancer cells results in the death of these cancers after bacterial infection. Loss of function of one or two IAP proteins among the eight members (12) is common in patients with malignancies. It was reported that somatic mutations of BIRC3 (cIAP2) were detected in 11% of splenic marginal zone lymphoma (71). BIRC2 (cIAP1) and BIRC3 are also frequently inactivated by copy number loss or by nonsense or frameshift mutations in multiple myeloma (72, 73). In addition, mutations of both *BIRC2* and *3* are also present in a wide range of epithelial tumors (74). These mutations result in the deficiency of their RING finger domain and loss of ubiquitin ligase activity being essential for transformation irrespective of NF-κB regulation (74). However, loss of function of one or two IAP members would not result in a cancer regression after an infection because IAPs can compensate for one another (75, 76). For example, deletion of XIAP results in increased cIAP1 and cIAP2 (75). Loss of function of multiple IAP members at the same time would be rare, like an infection induction of spontaneous regression of malignancies is rare. It was also reported that the nitric oxide (NO) donor glyceryl trinitrate (GTN) converts TNFα into a prodeath mediator in the colon and mammary cancer cells by inducing *S*-nitrosylation of cIAP1 (77). It is worth to further investigate if cancer with loss of function or structural modification of IAP proteins would be eliminated at the insidious stage by a bacterial induction of TNFα in the body.

Our findings in this report are of great significance to guide future clinical trials using IAP antagonists to treat cancers. For example, antibiotics can kill microbiota to reduce the level of LPS and the related TNFα and thus could attenuate the response of cancer patients to an IAP antagonist therapy. Therefore, antibiotics should not be used together with an IAP antagonist for cancer therapy in future clinical trials. National guidelines recommend one dose of perioperative antibiotics for breast surgery to prevent skin and soft tissue infection (78). In fact, many surgeons prescribe postoperative prophylactic antibiotics beyond the 24-h postoperative period in the setting of no reconstruction (79). The duration of prophylactic antibiotics can be 2–7 days, while some surgeons elect to continue antibiotics until all drains are removed (79). We recommend that IAP antagonist therapy should be postponed to the condition that host microbiota and levels of serum TNFα and LPS are restored to normal for those cancer patients who take antibiotic therapy.

Our findings would provide a useful inspiration to further investigate the mechanism action of BCG in treating bladder cancer. As a TLR4/2 agonist (80), BCG mediates MyD88-dependent innate immune response in the tumor microenvironment, contributing to the therapy of bladder cancer (81). However, it is not known if BCG, like LPS (**Figures 4**, **5**), activates MyD88 to stabilize IAP proteins in bladder cancer cells and if the subsequent increase of IAP proteins contributes to the failure of BCG in treating some cases of bladder cancer. It was reported that human bladder cancer tissues highly express several members of IAP proteins, including Survivin, cIAP1, cIAP2, XIAP, and Livin, which is an indicator of poor prognosis of bladder cancer (82). It is believed that BCG stimulation of TNFα production by either bladder cancer cells or host immune cells could trigger the apoptosis of bladder cancer when an IAP antagonist is given to degrade the IAP proteins. Thus, it is worth to further test if IAP antagonist can enhance the effect of BCG in treating bladder cancer, especially for those BCG non-responders.

Together, our findings previously (28) and in this report suggest that TNFα is essential for an IAP antagonist to treat cancer by inducing cancer cell apoptosis rapidly, and bacterial component LPS can also induce breast cancer cell apoptosis through TLR4 stimulating TNFα production by the host cells. A minimal level of LPS derived from microbiota in the body in the normal condition can stimulate TNFα production by host cells, in particular, macrophage (50, 57) and T lymphocyte (60). Therefore, antibiotics that kill microbiota to reduce the level of LPS and the related TNFα should not be used together with an IAP antagonist for cancer therapy. The low level of TNFα in patients could be a reason for the poor outcome of a single IAP antagonist in treating cancer in previous clinical trials (19–24). Our findings suggest that a combination of SM-164 with systemic administration of TNFα or LPS could be an effective therapy for advanced breast cancers including those with metastases. However, TNFα or LPS can result in serious side effects, including systemic shock, widespread inflammatory responses, and even death (35), due to its cytotoxic, cytostatic, and immunomodulatory properties (83–85). There is a strong rationale to develop small molecular compounds that mimic LPS or TNFα signaling to treat cancer in combination with an IAP antagonist while avoiding the serious side effects from LPS or TNFα. For example, LPS agonist, lipid A, has much less endotoxic than LPS and has been used as a vaccine adjuvant (86). It is worth to test if lipid A can be combined with an IAP antagonist for the treatment of breast and other cancers, including those relapsed and metastatic cancers.

## Data availability statement

The original contributions presented in the study are included in the article/**Supplementary Material**. Further inquiries can be directed to the corresponding author.

## Ethics statement

This study was reviewed and approved by University of Rochester Committee for Animal Resources.

## Author contributions

Conceptualization: ZY. Methodology: XL, JY, WL, and RD. Investigation: ZY, XL, JY, WL, RD, and ZC. Visualization: ZY. Supervision: ZY. Writing—original draft: ZY and JY. Writing—review and editing: ZY and JY. All authors contributed to the article and approved the submitted version.

## Funding

The research was supported by New York State Department of Health Breast Cancer Research Project C34928GG, U.S. Department of Defense Breast Cancer Research Program Award No W81XWH-22-1-0049, Breast Cancer Coalition of Rochester, and National Institutes of Health Grant R01AG049994 and R01AG076731. The opinions, interpretations, conclusions, and recommendations are those of the author and are not necessarily endorsed by the funding sources.

## Acknowledgments

Dr. Kamal U. Saikh at the Department of Immunology, Army Medical Research Institute of Infectious Diseases, Frederick, MD, USA, provided MyD88 inhibitor 4210. We sincerely thank the Core Services at the Center for Musculoskeletal Research (CMSR) (NIH/NIAMS P30 AR069655).

## Conflict of interest

The authors declare that the research was conducted in the absence of any commercial or financial relationships that could be construed as a potential conflict of interest.

## Publisher’s note

All claims expressed in this article are solely those of the authors and do not necessarily represent those of their affiliated organizations, or those of the publisher, the editors and the reviewers. Any product that may be evaluated in this article, or claim that may be made by its manufacturer, is not guaranteed or endorsed by the publisher.
