# Supplementary material for: Lipopolysaccharide sensitizes the therapeutic response of breast cancer to IAP antagonist
Source: Front Immunol. 2022 Aug 31;13:906357. doi: 10.3389/fimmu.2022.906357 (PMC9471085; doi:10.3389/fimmu.2022.906357)
Supplement: Supplementary file 1 [file DataSheet_1.doc]

Supplementary Material

**Supplementary Figure 1.** LPS is distinct to trigger the apoptosis of MDA-MB-231 cells in the presence of an IAP antagonist.

**
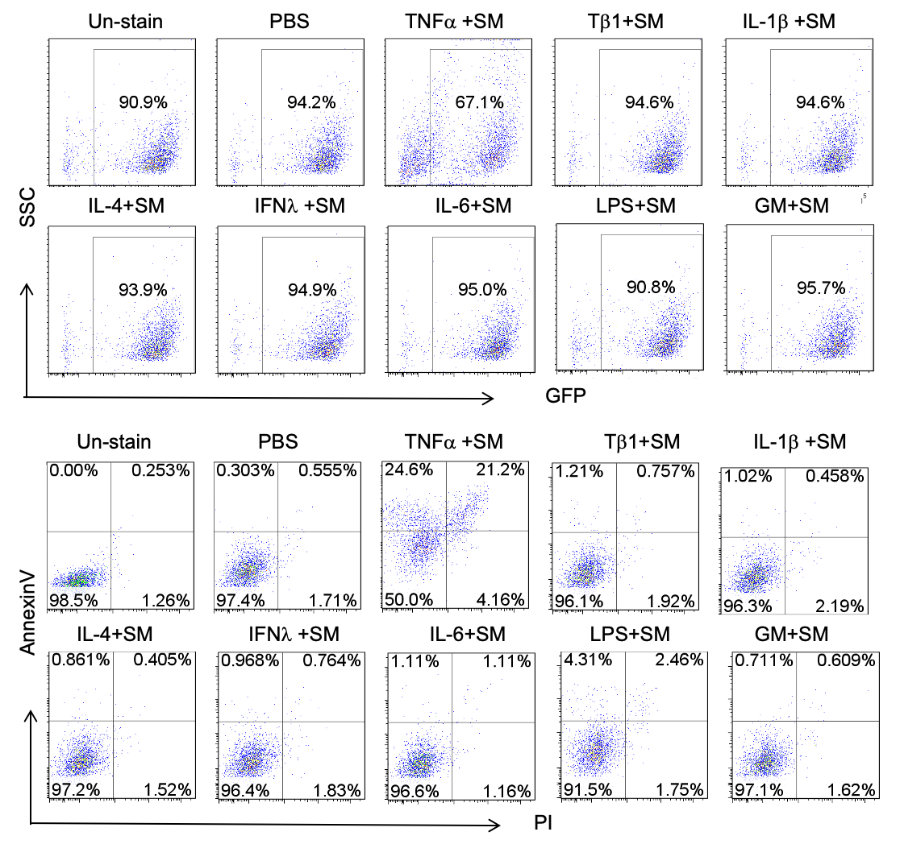
**

2x10^4^ of GFP^+^ MDA-MB231 cells were cultured in 60 mm dish for 24 hours followed by treatment over-night with 3 nM of SM-164 (SM) plus 30 ng/ml of LPS, 1 ng/ml of TNFα or 10 ng/ml of other cytokines, TGFβ1 (Tβ1), IL-1β, IL-4, IFNλ, IL-6, GM-CSF (GM). GFP^+^ live cells were gated (upper panel) to analyze the % of AnnV^+^PI^-/+^ apoptotic cells (lower panel) by flow cytometry. The experiments were repeated 3 times with similar results.

**Supplementary Figure 2.** LPS stimulates TNFα production by MDA-MB-231 cells via MyD88.

| 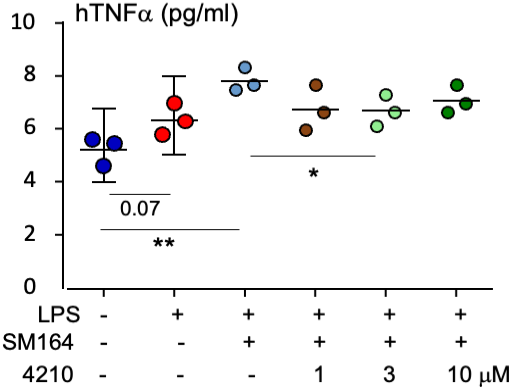 |
| --- |

2x10^4^ of MDA-MB-231 cells were cultured in 60 mm dish for 24 hours. The cells were then treated overnight with vehicle, SM164 (3 nM), LPS (100 ng/ml) and their combination with indicated dose of MyD88 inhibitor 4210. The culture medium was collected to test TNFα levels were tested by ELISA. The data were from 3 repeats. * p<0.05 and ** p<0.01 between the indicated groups.
